# Influence of Organic Nitrogen Derived from Recycled Wine Lees and Inorganic Nitrogen on the Chemical Composition of Cabernet Sauvignon Wines Fermented in the Presence of Non-Saccharomyces Yeasts *Candida boidinii*, *C. oleophila*, and *C. zemplinina*

**DOI:** 10.3390/foods13244166

**Published:** 2024-12-23

**Authors:** Claudia López-Lira, Pedro Valencia, Alejandra Urtubia, Esteban Landaeta, Ricardo A. Tapia, Wendy Franco

**Affiliations:** 1 Departamento de Química y Bioprocesos, Pontificia Universidad Católica de Chile, Av. Vicuña Mackenna 4860, Santiago 7820244, Chile; wfranco@uc.cl; 2 Centro de Investigación Daniel Alkalay Lowitt, Universidad Técnico Federico Santa María, Av. España 1680, Valparaíso 2390123, Chile; pedro.valencia@usm.cl; 3 Departamento de Ingeniería Química Medio Ambiental, Universidad Técnico Federico Santa María, Av. España1680, Valparaíso 2390123, Chile; alejandra.urtubia@usm.cl; 4 Escuela de Ingeniería, Universidad Central de Chile, Av. Santa Isabel 1186, Santiago 8330563, Chile; ealandaeta@uc.cl; 5 Facultad de Química y Farmacia, Pontificia Universidad Católica de Chile, Av. Vicuña Mackenna 4860, Santiago 6094411, Chile; rtapia@uc.cl; 6 Departamento de Ciencias de la Salud, Nutrición y Dietética, Pontificia Universidad Católica de Chile, Av. Vicuña Mackenna 4860, Santiago 7820244, Chile

**Keywords:** nonconventional yeast, volatile compounds, wine aroma, organic nitrogen, enzymatic hydrolysis, circular economy

## Abstract

In this study, the influences of inorganic nitrogen source (INS) and organic nitrogen source (ONS) supplementation during the wine fermentation process using three non-Saccharomyces yeasts (*Candida zemplinina*, *Candida oleophila*, and *Candida boidinii*) were analyzed. Diamine phosphate (DAP) was used as an INS, and lees enzymatic hydrolysate was used as an ONS. Complete alcoholic fermentation and a higher concentration of volatile compounds were obtained in fermentations with ONS, mainly esters from 81 to 4564 µg/L, alcohols from 231 to 7294 µg/L, and isoamyl acetate ester compounds from 12.3–22.8 ppb, with a very marked odorant activity value (OAV). In addition, malic acid was detected due to its influence on yeast metabolism and, consequently, on aroma production. Using a Y15 enzymatic autoanalyzer, residues of 1.30 g/L in ONS and 1.35 g/L in INS were obtained on the last day of alcoholic fermentation. In summary, we obtained promising results concerning the production of wine with enhanced functionalities due to higher concentrations of some volatile and polyphenolic compounds.

## 1. Introduction

Wine is a highly consumed beverage, and it is valued for its diverse aromas and flavors. These sensory characteristics are produced with the fruitful participation of yeasts during the grape juice fermentation process, where sugar and nitrogen play fundamental roles not only in ethanol production but also in the formation of the compounds responsible for most aromas in wine [1]. Nitrogen availability is essential to avoid slow or stuck fermentation, and nitrogen deficiency leads to slow fermentation kinetics and lower biomass yields. Furthermore, both the source and concentration of nitrogen have a direct effect on the number of volatile compounds, which are essential for wine flavor, the most predominant being acetate and ethyl esters, higher alcohols, and medium-chain and long-chain fatty acids [2,3,4,5,6].

Nitrogen is naturally present in grape juice as ammonium ions, amino acids, peptides, and proteins and is usually called yeast assimilable nitrogen (YAN). However, complete fermentation is frequently necessary to supplement nitrogen sources to reach an optimal concentration.

The wine industry widely uses inorganic nitrogen sources (INS), such as diammonium phosphate (DAP), to achieve the required nitrogen levels. However, INS increases the formation of acetic acid, which, in excessive quantities, gives the wine an acrid taste and an unpleasant vinegar aroma [7,8,9] On the other hand, studies have been conducted on the impact of using an organic nitrogen source (ONS), often primary amino acids, on the sensory profile of fermented wines, resulting in a decrease in acetic acid as well as improvements in other qualities, such as ethyl acetate esters and alcohol concentrations [10].

It has been reported that a 250 mg/L initial yeast assimilable nitrogen (YAN) concentration in grapes is adequate for an efficient fermentation process, but when it is less than 140 mg/L [11], nitrogen supplementation is important to avoid slow or stuck fermentation. In addition to the adequate control of YAN, other variables that could affect the organoleptic characteristics of wine are the nature of the yeast, pH, grape variety, and vinicultural practices [7,8,9]. The pH affects growth and fermentation performance; therefore, adequate control of the pH is necessary for obtaining high-quality wine. A pH of 2.9 inhibits the growth of microorganisms, affecting lactic acid bacteria culture inoculation and delaying alcoholic fermentation [12]. However, when the grape juice is in the 3.0–3.5 pH range, there is a lower chance of microbial damage and a greater chance of yeast growth, as described by [13].

Alcoholic fermentation has been traditionally achieved via the use of *Saccharomyces cerevisiae*, a yeast highly relevant in wine production [14]. Recently, increased grape sugar levels, likely driven by global warming, have led to an increased amount of alcohol in wine, which has become an important problem in the wine industry [15]. Therefore, the development of new fermentation processes enabling the production of wines with reduced alcohol content and maintained wine quality is currently a valuable objective. Interestingly, many studies have suggested that the use of non-Saccharomyces (NS) yeasts is suitable for reducing the alcohol content of wines [16]. Moreover, NS yeast improves specific parameters of wine quality [17]. However, the use of NS yeasts usually results in low fermentation performance, and these yeasts are traditionally utilized in combination with highly fermentative *S. cerevisiae* strains. As a result, interest in using NS yeast in mixed or sequential fermentations to produce wines with reduced alcohol content and enhanced organoleptic profiles has increased in recent decades [18,19,20]. Currently, Candida species have received increased attention for their ability to decrease the alcohol level and improve the aromatic profile of wines. For example, fermentation of mixed cultures of *S. cerevisiae* and *Candida zemplinina* increases glycerol production, generating softness and viscosity in the perception of the structure/body of the wine and decreasing the concentrations of alcohol, acetic acid, and fatty acids [21,22]. More recently, two indigenous Candida yeasts (*C. oleophila* and *C. boidinii*) showed interesting results in monoculture and sequential fermentations for reducing the ethanol concentration in Chilean Sauvignon Blanc Wines [23].

On the other hand, the determination of wine characteristics is very important and is usually achieved via chemical analysis. The most commonly used techniques for wine analysis are mass spectrometry (MS), UV-VIS spectroscopy, nuclear magnetic resonance, gas chromatography (GC), high-performance liquid chromatography (HPLC), GC-MS [24], and HPLC-MS, among others. However, these standard methods are generally expensive and difficult to execute, so it is often necessary to use a technique in which the sample does not have to be manipulated excessively. In this respect, a technique that has advantages over conventional ones is IR spectroscopy, which, in combination with multivariate analysis techniques, has been extensively used in the food industry and for quality control. Nevertheless, this technique is difficult to use when the samples contain water, so many of the characteristic bands of wine can be masked by the large absorption band of water at the time of analysis [25,26]. Considering the current analysis techniques and our recent results on the use of yeast protein hydrolysate (YPH) as a nitrogen source for grape must fermentation [27], we explored the potential of YPH as an organic nitrogen source for wine production using three Candida species, *C. boidinii*, *C. oleophila* and *C. zemplinina*. The study of the interaction of nonconventional yeasts with YPH for the production of a world-renowned beverage through biosustainable alternatives is very interesting considering the sensory differences of wine when it is treated with ONS [28]. The products of these different fermentations were characterized by high-performance liquid chromatography, autoanalyzer enzymatic Y15, and aromatic profiling with a technical gas chromatograph coupled to a mass detector (GCMS).

The various techniques used to characterize the wines as a final product allowed us to observe the ability of Candida yeasts to carry out complete fermentation in monocultures. These yeasts effectively assimilate different nitrogen sources, making them promising options for producing wines with distinctive aromatic qualities and lower ethanol levels while also promoting the reuse of wine industry byproducts as an environmentally friendly mitigation strategy.

## 2. Materials and Methods

### 2.1. Chemicals

Agarose seakem LE (Genexpress, Santiago, Chile), ammonium dihydrogen phosphate (Merck, Burlington, Germany), chloramphenicol agar medium (Condalab, Madrid, Spain), elution buffer (ThermoScientific, Rockford, United State), enzyme alcalase 2.4 L FG (Novozymes, Bagsvaerd, Denmark ), 10X TBE electrophoresis buffer (ThermoScientific, Rockford, United State), hydrochloric acid (Merck, Darmstadt, Germany), sodium hydroxide (Merck, Darmstadt, Germany), Sabouraud-2% dextrose broth (Condalab, Madrid, Spain), L (+)-tartaric acid (Merck, Darmstadt, Germany), and Milli-Q water (Merck, Darmstadt, GermanyMerck) were used. 

### 2.2. Yeast Strains

Three non-Saccharomyces yeast strains previously isolated from spontaneous fermentation of Carménère (*Candida zemplinina* LEVCZ, OP923900), Chardonnay (*Candida oleophila*, LEVCO, OP923900), and Merlot (*Candida boidinii*, LEVCB, OP923895) grape juices [29] were used. The yeasts were cultured at 28 °C for 24 h in Sabouraud dextrose broth. An inoculum of 10^6^ [CFU/mL] was used for fermentation.

### 2.3. Preparation of YPH as an Organic Nitrogen Source

A mixture of wine lees obtained from the Los Robles Emiliana winery, Chile, was used: Cabernet Sauvignon (14%), Carménère (45%) and Syrah (42%). This mixture was filtered, and the solid was treated with hydrochloric acid (0.361 L HCl/kg solid) for 45 min at 50 °C to dissolve the tartaric salts and then centrifuged for 3 min at 4000 rpm. The solid was reserved for YPH, and after evaporation and crystallization, it contained tartaric acid in 36% yield [30]. Then, the solid isolated previously was suspended in water at a 1:1 ratio, the pH was adjusted to 8 with 1.5 N NaOH, and an enzymatic hydrolysis reaction was carried out using Alcalase protease 2.4 L FG enzyme (15 mAU/g), manufactured by Novoenzymes (Bagsvaerd, Denmark), for 1 h at 50 °C. The hydrolysis reaction was finished by inactivating the enzyme at 85 °C for 30 min. The resulting YPH (10% grade) was used as ONS. The organic nitrogen content was determined via the Kjeldahal method (AOAC). DAP is used as an alternative nitrogen source since it is a chemical commonly used in the wine industry.

### 2.4. Fermentation Conditions

Fermentation for vinification at the laboratory scale was carried out in triplicate with Cabernet Sauvignon must from the 2021 season (Tarapacá Winery, Santiago, Chile). To obtain the must, the grapes were destemmed, crushed, pressed, and filtered to eliminate large particulates. Seventy ppm of sodium metabisulfite were added to the obtained grape juice. The grape juice had a density of 1.101 g/mL, 24.1 °Brix, and pH 4.1. The nitrogen content determined with a Y15 enzymatic autoanalyzer (Biosystems SA, Barcelona, Spain) was 207 mg/L. The pH was adjusted to 3.5 with L (+)-tartaric acid, and the nitrogen content was adjusted to 250 mg/L with DAP (43 mg/L) or YPH (43 mg/L). Finally, the yeast concentration was adjusted to approximately 1 × 10^6^ cells/mL. Then, fermentation monocultures of the must with each yeast, *C. boidinii* (Cb), *C. oleophila* (Co) and *C. zemplinina* (Cz), were carried out in triplicate in a 1000 mL Schott flask, which had an air lock and constant stirring at 100 rpm in a shaker (JSSI-100C, Gongju- Yes, Korea) at 27 °C. The fermentation processes were carried out for 9–11 days, and basic variables such as density, °Brix, and pH were measured to ensure complete alcoholic fermentation. All alcoholic fermentations carried out with the 3 species of Candida were left for 1 month for the decantation of yeasts and remaining organic material. After this process, the liquids were transferred to new Schott bottles and left for 2 more months for the precipitation of tartrates and malolactic fermentation (MLF) at a room temperature of 21 °C. At the end of this process, the wines were bottled and stored for analysis.

### 2.5. Analysis of Alcohols, Sugars, and Organic Acids

The analysis of individual alcohols, sugars, and organic acids was performed via high-performance liquid chromatography (HPLC) on an Agilent Model 1260 Infinity HPLC, manufactured by Agilent Technologies in Waldbronn, Germany, under isocratic conditions using a diode array (DAD), IR detectors and an Aminex HPX-87 chromatography column 300 × 7.8 mm, 9 μm particle size (Bio-Rad, Hercules, CA, USA). The mobile phase was 0.005 mM H_2_SO_4_, the flow rate was 0.6 mL/min, and the injection volume was 20 μL. Samples of the initial juice and from the last day of alcoholic fermentation were diluted with water (1:3 *v*/*v*), filtered (PVDF 13 mm/0.22 µm spin filters), and added to amber vials (2 mL) for analysis.

### 2.6. Analysis of the Aromatic Profile

An aromatic profile was carried out on the final wine samples through solid-phase microextraction and subsequent gas injection coupled to a mass detector. The chromatograms obtained were analyzed by comparing the mass spectra of the compounds found in each sample with those from the NIST-EPA-NIH library of 130,000 spectra. Each analysis was carried out in triplicate, and quantification was performed via the internal standard method, considering that a response factor equal to 1 GC 2010 Plus/GCMS QP2020 chromatograph was used.

The concentrations of volatile compounds, 7 acids, 26 alcohols, 4 aldehydes, 4 C6 compounds, 23 esters, 2 ketones, and 1 lactone, were quantified to characterize the bottled wines prepared with different nitrogen sources. The quantification was carried out via the internal standard method considering a response factor equal to 1. A total of 67 compounds were detected in samples of bottled wines for each yeast, Cb, Co, and Cz (supplemented with YPH or DAP).

### 2.7. Calculation of the Odor Activity Value (OAV)

The odor activity value (OAV) reflects the contribution of volatile compounds to wine aroma. The OAV was calculated for each volatile compound as the ratio between the total concentration Ci [microg/L] and its odor threshold value OTi according to the following formula:

OAV = Ci/OTi

Values greater than or equal to 1 represent a significant contribution to the aroma of the wine [31].

### 2.8. Data Analysis

Sampling and analysis were performed in triplicate, and the data are presented as the means ± SD. One-way analysis of variance (ANOVA) was conducted. Differences with a *p* value < 0.05 were considered significant. Tukey’s honestly significant difference (HSD) test was performed with Statistix 8, Analytical Software.

## 3. Results and Discussion

**Figure 1:**
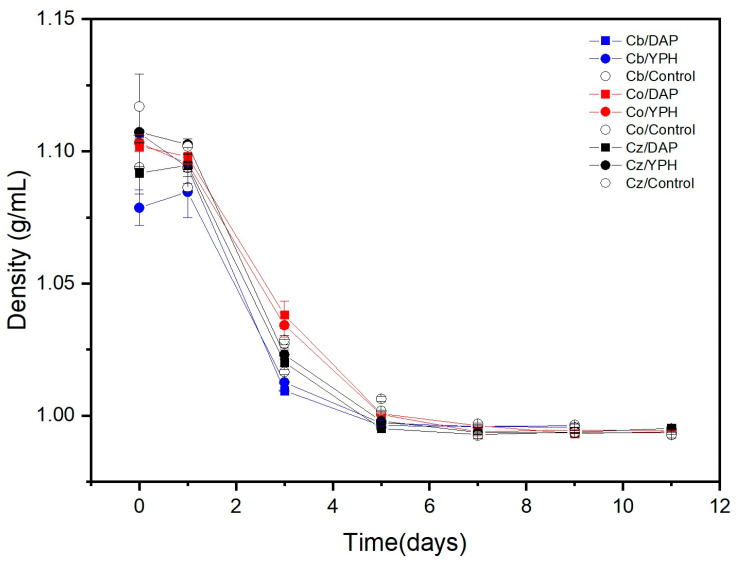
Fermentation kinetics of yeasts with different nitrogen sources: *C. boidinii* with DAP (Cb/DAP), *C. boidinii* with YPH (Cb/YPH), *C. oleophila* with DAP (Co/DAP), *C. oleophila* with YPH (Co/YPH), *C. zemplinina* with DAP (Cz/DAP), *C. zemplinina* with YPH (Cz/YPH) and controls with no nitrogen added: *C. boidinii* (Cb), *C. oleophila* (Co) and *C. zemplinina* (Cz).

Three indigenous non-Saccharomyces yeast strains, *C. boidinii* (Cb), *C. oleophila* (Co), and *C. zemplinina* (Cz), were evaluated for their enological potential to obtain red wine in monoculture fermentation using yeast protein hydrolysate (YPH) as the ONS and DAP as the INS. To ensure complete alcoholic fermentation, basic variables such as density, °Brix, and pH were measured. The fermentation process for *C. boidinii* was finished in 9 days, whereas *C. oleophila* and *C. zemplinina* fermentation required 11 days. The fermentation kinetics were observed by measuring the density of the must daily throughout the process until the end of fermentation. Here, Cb, Co, and Cz supplemented with DAP yielded average final densities of 0.995 g/mL: Cb, Co, and Cz with YPH 0.994 g/mL (Figure 1), with variations in acidity for Cb (3.6–3.7), Co (3.6–3.6) and Cz (3.6–3.9). The small effect of the addition of nitrogen on the kinetics during fermentation with *C. boidinii, C. oleophila* and *C. zemplinina* could be due to the high enough concentration of YAN in the initial must (207 mg/L) vs. the nitrogen concentration of 250 mg/L achieved with DAP/YPH supplementation. The successful alcoholic fermentation exhibited by these NS yeasts is an interesting result. A similar result has been described previously when *C. zemplinina*, *C. stellata*, *S. ludwigii*, and *D. bruxellensis* were used to produce white wines [32].

On the other hand, considering that NS yeasts can improve wine flavor and other sensory properties [1], investigating the influence of nitrogen on the aromatic profile of wines via *C. boidinii*, *C. oleophila* and *C. zemplinina* with the aim of providing insight into the metabolic mechanisms involved is important. For this purpose, the influence of nitrogen addition on the aromatic composition of the final wines was studied. First, the amounts of sugars, acids, and alcohols present in the fermented wines were determined via HPLC. The results, including those of the control without nitrogen addition, are shown in Table 1. The residual sugar content (glucose + fructose) did not differ between the yeast supplemented with DAP (INS) or YPH (ONS) and was less than 0.1 g/L, except in the control using *C. boidinii* (2.2 g/L), confirming the effective alcoholic fermentation by the NS yeasts tested. The ethanol concentration was slightly lower than that of the control, except for *C. zemplinina*. The lowest value was obtained for fermentation with *C*. *boidinii* supplemented with DAP (11.15% *v*/*v*), and the average value was 11.50% *v*/*v*. Previous studies revealed that the use of *C. boidinii* and *C. oleophila* yeasts can reduce the alcoholic content of Chilean Sauvignon Blanc wine [23]. Differences in sugar contents, such as glucose and fructose contents, directly affect the final ethanol concentration; however, today, the industry focuses on nonconventional yeasts because of their ability to obtain wine with reduced ethanol [33]. Furthermore, other factors, such as the assimilation of the nitrogen source by NS yeasts, biomass synthesis, metabolic flow and generation of a greater number of byproducts compared to SC, also influence the final ethanol yield. As expected for red wines [16,34], the concentration of glycerol reached significant levels, ranging from 8.61 to 10.40 g/L with DAP and YPH. Tartaric acid concentrations ranged from 1.56 g/L to 5.68 g/L, depending on the strain and conditions, whereas acetic acid levels varied from 0.08 g/L to 3.34 g/L. The ranges of tartaric acid are consistent with the typical levels found in grapes and wines reported in the literature, generally between 1.5 and 4 g/L [35]. Tartaric acid was correlated with astringency in wines. The values reported here indicate that this perception is similar to that of other wines. Acetic acid, an indicator of volatile acidity, is often found at concentrations below 1 g/L in well-controlled fermentations, although higher levels (above 1.5 g/L) can lead to negative sensory impacts, resulting in vinegar-like aromas [36]. In this study, acetic acid levels reached 3.34 g/L under certain conditions, which is above the sensory threshold of 0.7 g/L for wine and could significantly influence odor characteristics and reduce wine quality, imparting sourness and undesirable aromas. Conversely, the tartaric acid levels align with typical wine concentrations and contribute positively to the acid balance of wine, influencing freshness and stability. Thus, the analysis of the volatile profile of the wines indicated that the odorant capacity of the acid contained in the wine was less than 1, which suggests that it would not be perceived by the consumer [37,38]. Interestingly, all the fermentations supplemented with DAP produced less ethanol than did the control, with an average of 11.52% *v*/*v*. Similar results were obtained previously using *C. boidinii* and *C. oleophila* in Chilean Sauvignon Blanc wines [23]. The polyphenolic content of wine influences its organoleptic properties and therefore its quality.

**Figure 2:**
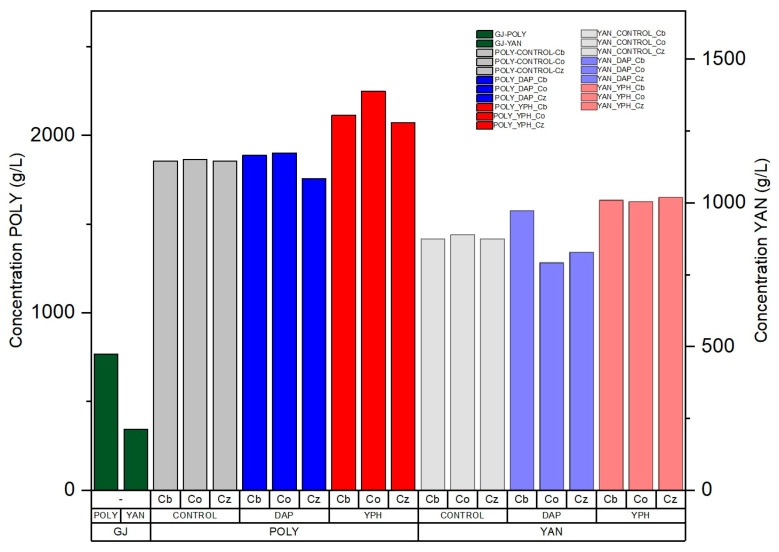
Characterization of polyphenols (POLY) and YAN in grape juice (GJ) before fermentation and characterization at the end of alcoholic fermentation with *C. boidinii* with DAP (DAP_Cb), *C. boidinii* with YPH (YPH_Cb), *C. oleophila* DAP (DAP_Co), *C. oleophila* with YPH (YPH_Co), *C. zemplinina* with DAP (DAP_Cz), *C. zemplinina* with YPH (YPH_Cz) and controls: *C. boidinii* (CONTROL_Cb), *C. oleophila* (CONTROL_Co) and *C. zemplinina* (CONTROL_Cz).

The results (Figure 2) revealed a significantly greater trend in the concentration of polyphenols (12–22%) at the end of fermentation in the YPH samples than in the DAP samples; additionally, a lower consumption of YAN was observed in the YPH samples. This result agrees with those previously reported for the fermentation of Cabernet Sauvignon grapes using *S. cerevisiae* [27]. Because one of the characteristics considered to define the quality of wine is color, the polyphenolic composition is important. The red–purple color given by anthocyanins can be affected by factors such as pH and the composition of the medium, where the latter generates reactions during the wine process that could affect the stability of polyphenols. Covinification has been reported as a methodology to improve the polyphenol content. Based on our results, the nitrogen content from lees favors this concentration because of its organic composition. Furthermore, the pH stability of Cb and Co would increase the stability of the polyphenols [38], thus favoring the qualities of the final wine.

Furthermore, the source of nitrogen available for yeast cell stimulation and increased biomass during the sugar consumption process in alcoholic fermentation is a factor that determines the organoleptic qualities of wine. In this context, amino acids are considered one of the most important sources during the alcoholic fermentation process, directly affecting yeast biomass and the production of volatile compounds. Some studies mention that biomass formation, as well as the type and quantity of volatile compounds, are largely dependent on individual amino acid concentrations [39] Therefore, analysis of polyphenolic concentrations and their relationships with different nitrogen sources was performed.

Esters, which are byproducts of alcoholic fermentation, are an important part of the aroma of a wine. Acetate esters can be produced by spontaneous esterification between acetate and alcohols mediated by acetyl-CoA enzymatic activity or by yeast enzymatic activity through the thioester acetyl-CoA, where the substrate of the alcohol acetyltransferase of the yeast cell has both acetyl-CoA and the corresponding alcohol. The formation of higher alcohols occurs via amino acid degradation through the Ehrlich pathway; however, higher alcohols could also be generated from glucose via pyruvate [40,41]. Based on these precedents, it is proposed that the increased bioavailability of nitrogen from amino acids in YPH could generate a higher concentration of alcohols, which would favor the formation of esters.

There are some reports that establish a relationship of some strains that, despite having a low nitrogen demand, generate high concentrations of 1-propanol, formed from the condensation of pyruvic acid and acetyl-CoA, according to results for both Cb and Co.

With respect to the source of organic nitrogen, it is possible to observe that, according to Table 1, there is less nitrogen consumption and a tendency toward a higher concentration of 1-propanol. However, for Cz/YPH, the same conditions are not maintained, which suggests that this trend would be affected by the type of nitrogen, as the amino acid content of the nitrogen source favors the production of alpha keto acids and consequently volatile alcohols [42].

Therefore, the aromatic profile was analyzed via GC/MS to determine the influence of nitrogen addition on the aromatic composition of the final wines. Higher concentrations of both alcohols and esters were obtained in alcoholic fermentation using *C. boidinii*, *C. oleophila* and *C. zemplinina* supplemented with YPH than with DAP. Isoamyl alcohol and esters were obtained at the highest concentrations [24] (Table 2) (Supplementary Materials S1–S6). An increase in the concentration of higher alcohols and esters might be advantageous for the quality of wine.

To determine the influence of the studied yeasts and nitrogen sources on the aroma of the final wine, the OAV with ONS and INS were determined. The OAV was calculated as the ratio between the concentration of the free volatile compound and its odor threshold. Table 3 shows the volatile compounds with an OAV > 1, and a complete list of the quantified volatile aroma compounds is shown in Table 3 (Supplementary Materials S7 and S8). The OAV measured for fermentation with *C. boidinii* were greater than those measured with *C. oleophila* and *C. zemplinina,* and isoamyl acetate represents one of the predominant aromatic compounds (sweet/fruity/banana/solvent/ester/pungent). Interestingly, fermentation with *C. oleophila* and *C. zemplinina* resulted in higher values with YPH than with DAP (Table 3). The compounds with the highest OAV were isoamyl acetate (21.9–22.8 μg/L, Cb), ethyl hexanoate (13.1–14.8, Cb), ethyl butanoate (7.2–8.0, Cb) and ethyl 3-methylbutanoate (6.3–7.6 Cb). Therefore, these esters are valuable contributors to the aroma and quality of the produced wines. Notably, an OAV greater than 1 is considered important at the odorant level in the total aromatic component of the sample with respect to the sensory threshold reported in the literature. As a result, in the wines fermented with *C. boidinii*, *C. oleophila* and *C. zemplinina*, greater odorant perception was detected in those samples with YPH. The characteristic aromas of these compounds are fruity, floral and banana aromas, which are characteristic of isoamyl fatty acids. Fruity aromas, characteristic of ethyl hexanoate, ethyl acetates and ethyl octanoate, prevail for compounds derived from medium-chain fatty acids such as ethyl esters [40,43].

Given that the metabolism of yeast and the supplied nitrogen play important roles in the formation of volatile compounds, malic acid also needs to be considered an essential molecule in the derivation of glyoxylate and the cycle of tricarboxylic acid and one that could be affected by the nitrogen source. It has been reported [44] that the use of YPH as a nitrogen source results in greater production of malate, the ionized form of malic acid that is easily metabolized by microorganisms.

The importance of the malic acid molecule is because it influences the metabolism of the yeast and therefore affects the production of aromas.

In addition, malic acid allows regulation of the final pH of wine. Currently, climate change has led to a problematic reduction in the concentration of malic acid in grapes during ripening, requiring the chemical addition of tartaric acid or malic acid to adjust the acidity; however, this is an expensive solution for the industry. Fermentation supplemented with YPH resulted in greater changes in malic acid at the end of the process, with Co and Cz being more significant in samples with *C. boidinii* (Table 4) [44,45].

The interest in malic acid and tartaric acid in the wine industry encouraged the search for methodologies that allow rapid identification and quantification.

## 4. Conclusions

In conclusion, through the reuse and post-fermentation treatments, we obtained wines with complete fermentation processes without stagnation and with different aromatic qualities when YPH was used. The final wines with YPH tended toward a higher concentration of volatile compounds such as alcohols and esters in the 3 species of Candida yeast analyzed. Additionally, a high concentration of isoamyl alcohol was generated, thus predominating Fusel, alcoholic, whiskey, fruity, banana, pungent, ether, cognac, and molasses aromas, with significant odorant activity from isoamyl acetate (sweet, fruity, banana, solvent, ester, pungent). Additionally, YPH generated a relatively high concentration of polyphenolic compounds at the end of alcoholic fermentation, which benefits from health-promoting properties such as antioxidant and cardioprotective properties, among others, which are widely reported.

Another quality observed in samples with YPH is that there is a greater remnant of malic acid in samples Co/YPH and Cz/YPH at the end of alcoholic fermentation, which would favor better pH regulation in the final wine sample. Considering the sensory differences of a wine when receiving treatment with ONS, the study of biosustainable alternatives to produce a beverage that is in high demand worldwide is very interesting.

## Figures and Tables

**Table 1:** Sugars, glycerol, tartaric acid, acetic acid, and ethanol analysis by HPLC of the fermentation assays with *C. boidinii*, *C. oleophila* and *C. zemplinina* supplemented with DAP (INS), YPH (ONS) and the control. Superscript letters a–f in the columns indicate significant differences (*p* < 0.05).

Yeast	Nitrogen Source	Glucose (g/L)	Fructose (g/L)	Glycerol (g/L)	Tartaric Acid (g/L)	Acetic Acid (g/L)	Ethanol (% *v*/*v*)
No yeast	Juice grape	63.40 ± 0.02	111.20 ± 0.02	0.96 ± 0.00	2.91 ± 0.01	2.76 ± 0.00	ND
*C. boidinii* Day 0	DAP (43 mg/L)	54.90 ± 0.02 ^e^	96.75 ± 0.01 ^d^	0.72 ± 0.00 ^d^	3.04 ± 0.00 ^cd^	1.40 ± 0.09 ^ab^	<0.02
	YPH (43 mg/L)	64.25 ± 0.01 ^cd^	112.50 ± 0.00 ^c^	0.78 ± 0.00 ^cd^	4.12 ± 0.00 ^bc^	1.50 ± 0.01 ^ab^	<0.02
	Control	63.72 ± 0.00 ^cd^	113.28 ± 0.08 ^c^	0.98 ± 0.00 ^bc^	4.19 ± 0.00 ^bc^	1.66 ± 0.00 ^a^	<0.02
*C. boidinii* Day 9	DAP (43 mg/L)	<0.10	<0.10	8.61 ± 0.00 ^d^	2.55 ± 0.02 ^a^	0.34 ± 0.02 ^d^	11.15 ± 0.00 ^c^
	YPH (43 mg/L)	<0.10	<0.10	9.20 ± 0.00 ^cd^	2.66 ± 0.00 ^a^	2.26 ± 0.01 ^b^	12.60 ± 0.01 ^b^
	Control	<0.10	2.20 ± 0.01	9.61 ± 0.00 ^bc^	1.76 ± 0.02 ^bc^	3.34 ± 0.01 ^a^	12.85 ± 0.01
*C. oleophila* Day 0	DAP (43 mg/L)	63.50 ± 0.12 ^d^	112.42 ± 0.03 ^c^	1.07 ± 0.00 ^ab^	4.33 ± 0.08 ^b^	1.28 ± 0.06 ^ab^	<0.02
	YPH (43 mg/L)	66.62 ± 0.10 ^b^	118.22 ± 1.88 ^b^	1.11 ± 0.00 ^ab^	4.56 ± 0.01 ^ab^	1.29 ± 0.00 ^ab^	<0.02
	Control	64.78 ± 0.09 ^c^	118.60 ± 0.00 ^b^	1.23 ± 0.00 ^a^	5.68 ± 0.22 ^a^	1.28 ± 0.00 ^ab^	<0.02
*C. oleophila* Day 11	DAP (43 mg/L)	<0.10	<0.10	10.14 ± 0.00 ^ab^	2.28 ± 0.00 ^ab^	0.49 ± 0.00 ^c^	11.91 ± 0.01 ^abc^
	YPH (43 mg/L)	<0.10	<0.10	10.09 ± 0.05 ^ab^	2.30 ± 0.01 ^ab^	<0.02	12.12 ± 0.04 ^abc^
	Control	<0.10	<0.10	10.58 ± 0.02 ^a^	2.35 ± 0.05 ^ab^	<0.02	12.69 ± 0.05 ^ab^
*C. zemplinina* Day 0	DAP (43 mg/L)	42.70 ± 0.02 ^f^	78.15 ± 0.25 ^e^	0.85 ± 0.02 ^cd^	2.26 ± 0.00 ^d^	1.03 ± 0.03 ^b^	<0.02
	YPH (43 mg/L)	67.44 ± 0.02 ^b^	118.21 ± 2.90 ^b^	1.14 ± 0.00 ^ab^	2.27 ± 0.33 ^d^	1.30 ± 0.00 ^ab^	<0.02
	Control	78.55 ± 0.41 ^a^	142.05 ± 3.13 ^a^	1.25 ± 0.02 ^a^	2.15 ± 0.00 ^d^	1.62 ± 0.06 ^a^	<0.02
*C. zemplinina* Day 11	DAP (43 mg/L)	<0.10	<0.10	9.29 ± 0.12 ^c^	1.56 ± 0.00 ^c^	0.08 ± 0.00 ^f^	11.41 ± 1.16 ^bc^
	YPH (43 mg/L)	<0.10	<0.10	10.40 ± 0.01 ^a^	1.48 ± 0.14 ^c^	0.36 ± 0.02 ^d^	13.32 ± 0.05 ^a^
	Control	<0.10	<0.10	10.27 ± 0.00 ^a^	1.48 ± 0.14 ^c^	0.27 ± 0.01 ^e^	13.03 ± 0.01 ^a^

**Table 2:** Concentration (µg/L) of alcohols and esters obtained from wine samples fermented with *C. boidinii*, *C. oleophila* and *C. zemplinina* yeasts and supplemented with 43 mg/L DAP (INS) or 43 mg/L YPH (ONS). Superscript letters a–f in the columns indicate significant differences (*p* < 0.05).

Compounds	Cb/DAP µg/L	Cb/YPH µg/L	Co/DAP µg/L	Co/YPH µg/L	Cz/DAP µg/L	Cz/YPH µg/L
Isoamyl alcohol	6430.91 ± 0.00 ^b^	7294.80 ± 0.00 ^a^	2589.34 ± 0.00 ^e^	2913.96 ± 0.00 ^c^	2365.57 ± 2.29 ^f^	2693.09 ± 0.00 ^d^
2-methylbutanol	2297.21 ± 0.00 ^b^	2455.46 ± 0.00 ^a^	529.52 ± 0.00 ^e^	834.86 ± 0.00 ^c^	518.29 ± 0.00 ^f^	782.12 ± 0.00 ^d^
Isobutyl alcohol	612.76 ± 0.00 ^b^	620.95 ± 0.00 ^a^	226.14 ± 0.00 ^f^	343.90 ± 2.90 ^c^	228.91 ± 0.00 ^e^	293.13 ± 0.00 ^d^
2-Phenyl ethanol	330.45 ± 4.23 ^b^	453.85 ± 0.00 ^a^	209.43 ± 0.00 ^e^	223.02 ± 0.00 ^d^	225.70 ± 0.00 ^d^	231.62 ± 0.00 ^c^
1-propanol	155.85 ± 0.00 ^b^	216.55 ± 3.11 ^a^	42.33 ± 3.00 ^d^	46.64 ± 1.16 ^cd^	48.80 ± 0.00 ^c^	33.37 ± 0.70 ^e^
2,3-butanediol Isomero 2	41.82 ± 0.00 ^b^	53.90 ± 0.00 ^a^	27.56 ± 0.00 ^c^	39.78 ± 0.00 ^b^	52.69 ± 2.92 ^a^	52.99 ± 0.00 ^a^
2,3-butanediol Isomero 1	16.90 ± 0.08 ^bc^	14.79 ± 0.86 ^cd^	12.63 ± 0.70 ^d^	14.14 ± 0.52 ^cd^	21.99 ± 0.30 ^a^	19.39 ± 0.76 ^ab^
Ethyl acetate	4816.51 ± 0.00 ^a^	4564.31 ± 0.00 ^b^	1252.76 ± 0.00 ^e^	1591.66 ± 0.00 ^c^	1209.30 ± 0.29 ^f^	1461.92 ± 0.00 ^d^
Ethyl hexanoate	812.85 ± 0.00 ^b^	917.11 ± 0.00 ^a^	275.58 ± 2.90 ^f^	506.46 ± 0.00 ^c^	299.56 ± 0.00 ^e^	459.03 ± 0.00 ^d^
Isoamyl acetate	657.70 ± 0.00 ^b^	684.50 ± 0.00 ^a^	289.75 ± 1.13 ^e^	298.90 ± 0.02 ^d^	248.05 ± 0.00 ^f^	369.00 ± 0.00 ^c^
Ethyl octanoate	639.59 ± 0.00 ^d^	678.22 ± 0.00 ^a^	290.14 ± 0.05 ^f^	667.76 ± 0.00 ^b^	387.80 ± 0.00 ^e^	642.09 ± 0.00 ^c^
Ethyl lactate	462.62 ± 0.00 ^b^	576.65 ± 0.00 ^a^	276.73 ± 3.23 ^f^	342.37 ± 0.00 ^d^	305.45 ± 0.00 ^e^	364.52 ± 0.00 ^c^
Ethyl butanoate	143.09 ± 0.00 ^b^	160.73 ± 0.00 ^a^	39.41 ± 0.97 ^cd^	41.52 ± 3.52 ^c^	38.15 ± 0.00 ^cd^	37.75 ± 0.39 ^d^
Diethyl butanedioate	130.58 ± 0.00 ^b^	136.45 ± 0.00 ^a^	93.33 ± 1.29 ^e^	103.26 ± 0.00 ^d^	95.30 ± 0.00 ^e^	117.28 ± 0.53 ^c^
Ethyl decanoate	36.10 ± 0.00 ^d^	41.80 ± 0.50 ^cd^	42.01 ± 8.04 ^c^	81.08 ± 8.51 ^a^	65.64 ± 0.00 ^b^	81.63 ± 0.00 ^a^

**Table 3:** Concentration of esters with the highest OAVs in wine samples fermented with *C. boidinii*, *C. oleophila* and *C. zemplinina* supplemented with DAP or YPH.

Compounds	Cb/DAP	Cb/YPH	Co/DAP	Co/YPH	Cz/DAP	Cz/YPH	OT
Ethyl 3-methylpentanoate	-	3.8	1.6	3.2	1.4	2.6	0.5
Ethyl hexanoate	13.1	14.8	4.4	8.2	4.8	7.4	62
Ethyl butanoate	7.2	8.0	2.0	2.1	1.9	1.9	20
Ethyl 2-methylbutanoate	1.0	1.2	0.1	0.1	0.2	0.2	18
Ethyl 3-methylbutanoate	6.3	7.6	0.7	0.9	0.7	0.8	3
Ethyl octanoate	1.1	1.2	0.5	1.2	0.7	1.1	580
Isoamyl acetate	21.9	22.8	9.7	10.0	8.3	12.3	30

**Table 4:** Quantification of malic acid at the beginning and end of alcoholic fermentation with supplemented *C. boidinii*, *C. oleophila* and *C. zemplinina* yeasts via an enzymatic autoanalyzer Y15 (Biosystems SA, Barcelona, Spain). Superscript letters a–g in the columns indicate significant differences (*p* < 0.05).

Yeast	Nitrogen Source	Malic Acid (g/L)	pH
No yeast	**Juice grape**	1.27 ± 0.00	3.62 ± 0.00
*C. boidinii* Day 0	**DAP (43 mg/L)**	1.26 ± 0.00 ^bc^	3.73 ± 0.00 ^a^
	**YPH (43 mg/L)**	1.23 ± 0.36 ^bc^	3.65 ± 0.00 ^ab^
	**Control**	1.24 ± 0.00 ^bc^	3.65 ± 0.01 ^ab^
*C. boidinii* Day 9	**DAP (43 mg/L)**	1.35 ± 0.00 ^a^	3.71 ± 0.00 ^a^
	**YPH (43 mg/L)**	1.13 ± 0.00 ^c^	3.66 ± 0.00 ^bc^
	**Control**	1.24 ± 0.00 ^b^	3.70 ± 0.00 ^ab^
*C. oleophila* Day 0	**DAP (43 mg/L)**	1.25 ± 0.00 ^b^	3.60 ± 0.00 ^ab^
	**YPH (43 mg/L)**	1.26 ± 0.00 ^bc^	3.62 ± 0.00 ^b^
	**Control**	1.23 ± 0.00 ^bc^	3.62 ± 0.00 ^b^
*C. oleophila* Day 11	**DAP (43 mg/L)**	0.03 ± 0.00 ^fg^	3.62 ± 0.00 ^c^
	**YPH (43 mg/L)**	0.08 ± 0.00 ^e^	3.63 ± 0.00 ^bc^
	**Control**	0.04 ± 0.00 ^fg^	3.64 ± 0.00 ^c^
*C. zemplinina* Day 0	**DAP (43 mg/L)**	1.15 ± 0.00 ^c^	3.63 ± 0.00 ^b^
	**YPH (43 mg/L)**	1.30 ± 0.00 ^b^	3.62 ± 0.00 ^b^
	**Control**	1.35 ± 0.00 ^a^	3.63 ± 0.00 ^b^
*C. zemplinina* Day 11	**DAP (43 mg/L)**	0.02 ± 0.00 ^g^	3.91 ± 0.00 ^c^
	**YPH (43 mg/L)**	0.32 ± 0.00 ^d^	3.91 ± 0.00 ^c^
	**Control**	0.04 ± 0.00 ^f^	3.91 ± 0.00 ^c^

## Data Availability

The original contributions presented in the study are included in the article, further inquiries can be directed to the corresponding author.

## Supplementary Materials

The following supporting information can be downloaded at: https://www.mdpi.com/article/10.3390/foods13244166/s1, Table S1: Concentration (µg/L) of Acids obtained from wine samples fermented with *C. boidini*, *C. oleophila* and *C. zemplinina* yeasts and supplemented with DAP 43 mg/L (INS) YPH 43 mg/L (ONS), Table S2: Concentration (µg/L) of Alcohols obtained from wine samples fermented with *C. boidini*, *C. oleophila* and *C. zemplinina* yeasts and supplemented with DAP 43 mg/L (INS) YPH 43 mg/L (ONS), Table S3: Concentration (µg/L) of Aldehydes obtained from wine samples fermented with *C. boidini*, *C. oleophila* and *C. zemplinina* yeasts and supplemented with DAP 43 mg/L (INS) YPH 43 mg/L (ONS),Table S4: Concentration (µg/L) of C6 compounds obtained from wine samples fermented with *C. boidini*, *C. oleophila* and *C. zemplinina* yeasts and supplemented with DAP 43 mg/L (INS) YPH 43 mg/L (ONS), Table S5: Concentration (µg/L) of Ketones obtained from wine samples fermented with *C. boidini*, *C. oleophila* and *C. zemplinina* yeasts and supplemented with DAP 43 mg/L (INS) YPH 43 mg/L (ONS), Table S6: Concentration (µg/L) of Esters obtained from wine samples fermented with *C. boidini*, *C. oleophila* and *C. zemplinina* yeasts and supplemented with DAP 43 mg/L (INS) YPH 43 mg/L (ONS), Table S7: Concentration of Acids, Alcohols, Aldehydes, C6 Compounds with highest OAV of wine samples fermented with *C. boidini*, *C. oleophila* and *C. zemplinina* supplemented with DAP or YPH, Table S8: Concentration of Esters, Lactones with highest OAV of wine samples fermented with *C. boidini*, *C. oleophila* and *C. zemplinina* supplemented with DAP or YPH.
