# Supplementary material for: Influence of Organic Nitrogen Derived from Recycled Wine Lees and Inorganic Nitrogen on the Chemical Composition of Cabernet Sauvignon Wines Fermented in the Presence of Non-Saccharomyces Yeasts Candida boidinii, C. oleophila, and C. zemplinina
Source: Foods. 2024 Dec 23;13(24):4166. doi: 10.3390/foods13244166 (PMC11675325; doi:10.3390/foods13244166)
Supplement: Supplementary file 1 [file foods-13-04166-s001.zip › foods-3205971-supplementary.pdf]

# Influence of Organic Nitrogen Derived from Recycled Wine Lees and Inorganic Nitrogen on the Chemical Composition of Cabernet Sauvignon Wines Fermented in the Presence of Non-Saccharomyces Yeasts *Candida boidinii*, *C. oleophila,* and *C. zemplinina*.

**Claudia López-Lira [1,*], Pedro Valencia [2], Alejandra Urtubia [3], Esteban Landaeta [4], Ricardo A. Tapia [5] and Wendy Franco [1,6]**

[1] Departamento de Química y Bioprocesos, Pontificia Universidad Católica de Chile, Av. Vicuña Mackenna 4860, Santiago 7820244, Chile; clopez6@uc.cl, wfranco@uc.cl
[2] Centro de Investigación Daniel Alkalay Lowitt, Universidad Técnico Federico Santa María, Av. España 1680, Valparaíso 2390123, Chile; pedro.valencia@usm.cl
[3] Departamento de Ingeniería Química Medio Ambiental, Universidad Técnico Federico Santa María, Av. España1680, Valparaíso 2390123, Chile; alejandra.urtubia@usm.cl
[4] Escuela de Ingeniería, Universidad Central, Av. Santa Isabel 1186, Santiago 8330563, Chile; ealandaeta@uc.cl
[5] Facultad de Química y Farmacia, Pontificia Universidad Católica de Chile, Av. Vicuña Mackenna 4860, Santiago 6094411, Chile; rtapia@uc.cl
[6] Departamento de Ciencias de la Salud, Nutrición y Dietética, Pontificia Universidad Católica de Chile, Av. Vicuña Mackenna 4860, Santiago 7820244, Chile; wfranco@uc.cl
* Correspondence: clopez6@uc.cl

| COMPOUNDS | Cb/DAP µg/L | Cb/YPH µg/L | Co/DAP µg/L | Co/YPH µg/L | Cz/DAP µg/L | Cz/YPH µg/L | OLFACTORY DESCRIPTOR |
|---|---|---|---|---|---|---|---|
| ***ACIDS*** | | | | | | | |
| 2-Methoxypropanoic acid | 10.9 | 11.4 | 7.2 | 4.0 | 8.1 | 3.7 | - |
| Acetic acid | 165.2 | 161.2 | 83.0 | 116.3 | 96.5 | 87.4 | Sharp / acrid / sour / vinegar |
| Butanoic acid | N.D. | N.D. | 7.8 | 9.4 | 8.6 | 7.9 | Sharp / acetic / cheese / butter / fruity / dairy |
| Decanoic acid | N.D. | N.D. | 5.9 | 7.6 | 6.6 | 5.3 | Rancid / sour / fatty / citrus |
| Hexanoic acid | 12.8 | 17.0 | 11.1 | 16.0 | 13.5 | 14.4 | - |
| Isovaleric acid | 9.2 | 12.8 | 2.2 | 2.2 | 2.1 | 2.5 | Sour / sweet / cheese / tropical / dairy / acid / acrid / fruity / fatty |
| Octanoic acid | 15.1 | 18.3 | 10.6 | 17.1 | 13.7 | 16.2 | fatty / wax / rancid / oil / vegetable / cheese / soup / brandy |
| **Total** | **213.2** | **220.7** | **127.7** | **172.5** | **149.1** | **137.4** | |

**Table S1:** Concentration (µg/L) of Acids obtained from wine samples fermented with *C. boidini, C. oleophila* and *C. zemplinina* yeasts and supplemented with DAP 43 mg/L (INS) YPH 43 mg/L (ONS).

| COMPOUNDS | Cb/DAP µg/L | Cb/YPH µg/L | Co/DAP µg/L | Co/YPH µg/L | Cz/DAP µg/L | Cz/YPH µg/L | OLFACTORY DESCRIPTOR |
|---|---|---|---|---|---|---|---|
| **ALCOHOLS** | | | | | | | |
| 1-Butanol | 19.0 | 20.1 | 5.3 | 5.3 | 5.7 | 5.5 | Fusel / oil/ sweet / balsamic / whiskey |
| 1-Decanol | 1.5 | 2.2 | 1.7 | 1.9 | 1.7 | 1.8 | Oily/ waxy / floral / orange / sweet / clean / watery |
| 1-Heptanol | 8.9 | 10.5 | 5.3 | 4.8 | 5.4 | 4.5 | Moldy / leafy / herbal / sweet / woody / peony / pungent / vegetal / fruity / apple / banana |
| 1-Nonanol | 2.6 | 2.7 | 3.4 | 2.9 | 4.3 | 4.0 | Fresh / clean / greasy / floral / rose / orange / oil / powder/ wet / wax / citrus / rue / aldehydic |
| 1-Octanol | 4.5 | 6.1 | 3.1 | 3.6 | 4.0 | 3.2 | Wax / orange / aldehyde / rose / mushroom / citrus / floral / sweet / greasy / coconut |
| 1-Pentanol | 3.4 | 3.2 | 1.7 | 1.9 | 1.6 | 1.7 | Fusel / oil / sweet / balsamic / pungent / fermented / bread / yeast / wine / solvent |
| 1-Propanol | 155.9 | 216.6 | 42.3 | 46.6 | 48.8 | 33.4 | Alcoholic / fermented / musty / fusel / tequila / yeast / sweet / fruity / apple / pear |
| 2,3-Butanediol Isomero 1 | 16.9 | 14.8 | 12.6 | 14.1 | 22.0 | 19.4 | Fruity / creamy / buttery |
| 2,3-Butanediol Isomero 2 | 41.8 | 53.9 | 27.6 | 39.8 | 52.7 | 53.0 | Fruity / creamy / buttery |
| 2-Ethyl-1-hexanol | 2.1 | 1.3 | 1.6 | 1.3 | 2.1 | 1.7 | Citrus / fresh / floral / oil / sweet |
| 2-Heptanol | 7.6 | 12.2 | 5.6 | 6.2 | 4.1 | 4.1 | Fresh / citronella / herbal / sweet / floral / fruity |
| 2-Methylbutanol | 2297.2 | 2455.5 | 529.5 | 834.9 | 518.3 | 782.1 | Ethereal / fermented |
| 2-Nonanol | N.D. | 5.8 | 5.5 | 6.7 | 4.4 | 3.9 | Waxy / creamy / citrus / orange / cheese / fruity |
| 2-Octenol | N.D. | N.D. | 0.4 | 0.5 | 0.6 | 0.4 | Green vegetable. |
| 3-Ethoxypropanol | N.D. | N.D. | 4.8 | N.D. | 4.6 | 0.4 | - |
| 3-Ethyl-4-methylpentan-1-ol | 6.4 | 5.5 | 4.6 | 5.4 | 5.4 | 5.6 | - |
| 3-Methyl-3-heptanol | N.D. | N.D. | 1.1 | N.D. | 0.4 | 2.1 | - |
| 3-Methylpentanol | 6.8 | 11.3 | 1.6 | 2.6 | 1.7 | 2.9 | Fusel / cognac / wine / cocoa / fruity / pungent |
| 3-Octanol | 3.3 | 4.4 | 2.8 | 4.0 | 3.0 | 4.1 | Earthy / mushroom / dairy / herbal / melon / citrus / woody / spicy / mint / musty / waxy / fermented |
| Glycerol | N.D. | 31.4 | 10.1 | 77.5 | 114.3 | 63.2 | - |
| Isoamyl alcohol | 6430.9 | 7294.8 | 2589.3 | 2914.0 | 2365.6 | 2693.1 | Fusel / alcohol / whiskey / fruity / banana / pungent / ethereal / cognac / molasses |
| Isobutyl alcohol | 612.8 | 621.0 | 226.1 | 343.9 | 228.9 | 293.1 | Ethereal / wine / bark |
| Isohexyl alcohol | 5.9 | 8.4 | 1.5 | 2.5 | 1.5 | 2.3 | Nut |
| Isopropenylethyl alcohol | 1.1 | 1.5 | 0.6 | 0.6 | 0.5 | 0.6 | sweet / fruity |
| 2-Phenyl ethanol | 330.5 | 453.9 | 209.4 | 223.0 | 225.7 | 231.6 | Floral / withered rose / sweet / fresh / bread / honey |
| Propylene Glycol | 4.4 | N.D. | 1.3 | 6.0 | 2.9 | 2.6 | Mild / alcohol |
| **Total** | **9963.5** | **11237.1** | **3698.8** | **4550.0** | **3630.2** | **4220.3** | |

**Table S2:** Concentration (µg/L) of Alcohols obtained from wine samples fermented with *C. boidini, C. oleophila* and *C. zemplinina* yeasts and supplemented with DAP 43 mg/L (INS) YPH 43 mg/L (ONS).

| COMPOUNDS | Cb/DAP µg/L | Cb/YPH µg/L | Co/DAP µg/L | Co/YPH µg/L | Cz/DAP µg/L | Cz/YPH µg/L | OLFACTORY DESCRIPTOR |
|---|---|---|---|---|---|---|---|
| *ALDEHYDES* | | | | | | | |
| Decanal | N.D. | N.D. | 0.3 | 0.3 | 0.3 | 0.2 | Sweet / aldehyde / wax / orange peel / citrus / floral |
| Hexanal | N.D. | N.D. | 0.4 | N.D. | 0.1 | 0.3 | Fresh / fatty / aldehyde / leaf / fruity / sweaty / vegetable / clean |
| Nonanal | N.D. | N.D. | 0.3 | N.D. | 0.6 | 0.5 | Wax / aldehyde / rose / fresh / lily /orange peel/ greasy |
| Octanal | N.D. | N.D. | 0.4 | N.D. | 0.6 | 0.3 | Aldehyde / wax / citrus / orange peel / greasy |
| **Total** | **N.D.** | **N.D.** | **1.5** | **0.3** | **1.6** | **1.3** | |

**Table S3:** Concentration (µg/L) of Aldehydes obtained from wine samples fermented with *C. boidini, C. oleophila* and *C. zemplinina* yeasts and supplemented with DAP 43 mg/L (INS) YPH 43 mg/L (ONS).

| COMPOUNDS | Cb/DAP µg/L | Cb/YPH µg/L | Co/DAP µg/L | Co/YPH µg/L | Cz/DAP µg/L | Cz/YPH µg/L | OLFACTORY DESCRIPTOR |
|---|---|---|---|---|---|---|---|
| *C6 COMPOUNDS* | | | | | | | |
| 1-Hexanol | 196.3 | 207.0 | 96.1 | 103.9 | 113.7 | 102.4 | Ethereal / fusel / oil / fruity / alcohol / sweet / pungent |
| E-3-Hexen-1-ol | N.D. | N.D. | 0.7 | 0.9 | 0.5 | 1.0 | Green bark / bush / leaf / floral / petal / oil / earth |
| Z-2-Hexen-1-ol | 1.6 | 1.4 | 1.0 | 1.2 | 0.8 | 1.1 | Fresh / green leaf / fruity / vegetable / greasy/ green beans / fusel / alcohol / whiskey |
| Z-3-Hexen-1-ol | 2.8 | 2.5 | 1.6 | 1.9 | 1.8 | 2.0 | Fresh / greasy / green foliage / herbal / oil / cut grass / melon peel / pungent |
| **Total** | **200.7** | **211.0** | **99.5** | **107.9** | **116.9** | **106.5** | |

**Table S4:** Concentration (µg/L) of C6 compounds obtained from wine samples fermented with *C. boidini, C. oleophila* and *C. zemplinina* yeasts and supplemented with DAP 43 mg/L (INS) YPH 43 mg/L (ONS).

| COMPOUNDS | Cb/DAP µg/L | Cb/YPH µg/L | Co/DAP µg/L | Co/YPH µg/L | Cz/DAP µg/L | Cz/YPH µg/L | OLFACTORY DESCRIPTOR |
|---|---|---|---|---|---|---|---|
| *KETONES* | | | | | | | |
| 1-Ethoxy-4,4-dimethyl-2-pentanone | 2.8 | 2.7 | 1.0 | 1.1 | 0.8 | 0.9 | - |
| **Total** | **2.8** | **2.7** | **1.0** | **1.1** | **0.8** | **0.9** | |
| *Lactones* | | | | | | | |
| Butyryl lactone | 6.0 | 8.6 | 2.5 | 2.4 | 2.6 | 2.4 | Creamy / oil / greasy / caramel |
| **Total** | **6.0** | **8.6** | **2.5** | **2.4** | **2.6** | **2.4** | |

**Table S5:** Concentration (µg/L) of Ketones obtained from wine samples fermented with *C. boidini, C. oleophila* and *C. zemplinina* yeasts and supplemented with DAP 43 mg/L (INS) YPH 43 mg/L (ONS).

| COMPOUNDS | Cb/DAP µg/L | Cb/YPH µg/L | Co/DAP µg/L | Co/YPH µg/L | Cz/DAP µg/L | Cz/YPH µg/L | OLFACTORY DESCRIPTOR |
|---|---|---|---|---|---|---|---|
| *Esters* | | | | | | | |
| Diethyl butanedioate | 130.6 | 136.5 | 93.3 | 103.3 | 95.3 | 117.3 | fruity / cooked apple / Cananga flower |
| Ethyl 2-hydroxyisovalerate | N.D. | N.D. | 1.2 | N.D. | 0.7 | 2.1 | - |
| Ethyl 2-methylbutanoate | 18.4 | 21.5 | 3.0 | 2.5 | 2.9 | 2.1 | Sharp / sweet / green apple / fruity / ester / berry / fresh /tropical |
| Ethyl 3-hexenoate | N.D. | N.D. | 0.7 | 1.0 | 0.4 | 1.1 | Sweet / metallic/ tropical/ rhubarb /weedy |
| Ethyl 3-methylbutanoate | 19.0 | 22.8 | 2.5 | 2.6 | 2.2 | 2.2 | Fruity / sweet / apple / pineapple / tutti frutti / ester / sharp / green apple/ orange |
| Ethyl 3-methylpentanoate | N.D. | 1.9 | 0.8 | 1.6 | 0.7 | 1.3 | Pineapple / fruit / natural / tropical |
| Ethyl Acetate | 4816.5 | 4564.3 | 1252.8 | 1591.7 | 1209.3 | 1461.9 | Ethereal / fruity / sweet / weedy |
| Ethyl butanoate | 143.1 | 160.7 | 39.4 | 41.5 | 38.2 | 37.8 | Fruity / pineapple juice / cognac / sweet / tutti frutti |
| Ethyl decanoate | 36.1 | 41.8 | 42.0 | 81.1 | 65.6 | 81.6 | sweet/creamy /fruity/ apple/ grape/ oily/ brandy |
| Ethyl heptanoate | 4.7 | 4.8 | 4.0 | 4.5 | 4.2 | 4.9 | Fruity / pineapple / cognac / rum / wine / sweet / éster / banana / berry |
| Ethyl hexanoate | 812.9 | 917.1 | 275.6 | 506.5 | 299.6 | 459.0 | Sweet / fruity / pineapple / wax / banana / fat / ester |
| Ethyl isopentyl succinate | 5.0 | 5.1 | 7.2 | 6.4 | 9.5 | 7.4 | - |
| Ethyl lactate | 462.6 | 576.7 | 276.7 | 342.4 | 305.5 | 364.5 | Intense / sour / fruity / buttery / acidic / ethereal / butterscotch |
| Ethyl nonanoate | 0.4 | N.D. | 1.8 | 1.3 | 2.2 | 2.0 | Fruity / rose / wax / rum / wine / natural / tropical / ester / cognac / apple/ banana |
| Ethyl octanoate | 639.6 | 678.2 | 290.1 | 667.8 | 387.8 | 642.1 | Fruity/ wine / waxy / sweet / apricot / banana / brandy / pear / musty / pineapple / dairy/ creamy |
| Ethyl valerate | 2.0 | N.D. | 1.0 | 1.4 | 0.9 | 1.1 | Sweet / fruity / apple / pineapple/ tropical / sour / berry |
| Hexyl ethanoate | 5.4 | 4.5 | 2.5 | 3.4 | 3.1 | 2.3 | Fruity / green apple / banana / sweet / fatty/ fresh/ pear |
| Isoamyl acetate | 657.7 | 684.5 | 289.8 | 289.9 | 248.1 | 369.0 | Sweet / fruity / banana / solvent / ester / pungent |
| Isoamyl hexanoate | N.D. | N.D. | 1.6 | 2.8 | 2.0 | 3.0 | Fruity / banana / apple/ pineapple / pungent / sour / cheese |
| Isoamyl isobutanoate | 1.3 | 1.6 | 1.0 | 0.7 | 0.8 | 0.7 | Tropical/ grape green/ cherry/ banana /green apple/ cocoa |
| Isoamyl lactate | 4.5 | 6.8 | 5.3 | 6.3 | 6.1 | 4.3 | Fruity / creamy/ nutty |
| Isobutyl acetate | 37.1 | 34.2 | 13.4 | 23.5 | 12.6 | 20.1 | sweet / fruity/ ethereal / banana / tropical / apple |
| Phenethyl acetate | 5.0 | 5.0 | 4.2 | 3.5 | 4.5 | 4.1 | Floral / rose / sweet / honey / fruity / tropical / yeast / cocoa / balsamic |
| **Total** | **7801.9** | **7868.0** | **2609.9** | **3685.7** | **2702.2** | **3591.9** | |

**Table S6:** Concentration (µg/L) of Esters obtained from wine samples fermented with *C. boidini, C. oleophila* and *C. zemplinina* yeasts and supplemented with DAP 43 mg/L (INS) YPH 43 mg/L (ONS).

| COMPOUNDS | ODOR THRESHOLD (ppb) | Cb/DAP µg OAV** | Cb/YPH µg/L OAV** | Co/DAP µg/L OAV** | Co/YPH µg/L OAV** | Cz/DAP µg/L OAV** | Cz/YPH µg/L OAV** |
|---|---|---|---|---|---|---|---|
| ***Acids*** | | | | | | | |
| Acetic acid | 300000 | 0.0 | 0.0 | 0.0 | 0.0 | 0.0 | 0.0 |
| Butanoic acid | 173 | - | 0.0 | 0.0 | 0.1 | 0.0 | 0.0 |
| Decanoic acid | 1000 | - | 0.0 | 0.0 | 0.0 | 0.0 | 0.0 |
| Isovaleric acid | 33 | 0.3 | 0.1 | 0.1 | 0.1 | 0.1 | 0.1 |
| Octanoic acid | 500 | 0.0 | 0.0 | 0.0 | 0.0 | 0.0 | 0.0 |
| ***Alcohols*** | | | | | | | |
| 1-Butanol | 150000 | 0.0 | 0.0 | 0.0 | 0.0 | 0.0 | 0.0 |
| 1-Decanol | 400 | 0.0 | 0.0 | 0.0 | 0.0 | 0.0 | 0.0 |
| 1-Heptanol | 1000 | 0.0 | 0.0 | 0.0 | 0.0 | 0.0 | 0.0 |
| 1-Octanol | 900 | 0.0 | 0.0 | 0.0 | 0.0 | 0.0 | 0.0 |
| 1-Pentanol | 64000 | 0.0 | 0.0 | 0.0 | 0.0 | 0.0 | 0.0 |
| 1-Propanol | 50000 | 0.0 | 0.0 | 0.0 | 0.0 | 0.0 | 0.0 |
| 2,3-Butanediol Isomero 1 | 150000 | 0.0 | 0.0 | 0.0 | 0.0 | 0.0 | 0.0 |
| 2,3-Butanediol Isomero 2 | 150000 | 0.0 | 0.0 | 0.0 | 0.0 | 0.0 | 0.0 |
| 2-Ethyl-1-hexanol | 8000 | 0.0 | 0.0 | 0.0 | 0.0 | 0.0 | 0.0 |
| 2-Heptanol | 70 | 0.1 | 0.1 | 0.1 | 0.1 | 0.1 | 0.1 |
| 2-Nonanol | 58 | - | 0.1 | 0.1 | 0.1 | 0.1 | 0.1 |
| Isoamyl alcohol | 30000 | 0.2 | 0.1 | 0.1 | 0.1 | 0.1 | 0.1 |
| Isobutyl alcohol | 40000 | 0.0 | 0.0 | 0.0 | 0.0 | 0.0 | 0.0 |
| Isohexyl alcohol | 50000 | 0.0 | 0.0 | 0.0 | 0.0 | 0.0 | 0.0 |
| 2-Phenyl ethanol | 14000 | 0.0 | 0.0 | 0.0 | 0.0 | 0.0 | 0.0 |
| ***Aldehydes*** | | | | | | | |
| Decanal | 1000 | - | 0.0 | 0.0 | 0.0 | 0.0 | 0.0 |
| Hexanal | 4.5 | - | 0.1 | 0.1 | - | 0.0 | 0.1 |
| Nonanal | 15 | - | 0.0 | 0.0 | - | 0.0 | 0.0 |
| ***C6 Compounds*** | | | | | | | |
| 1-Hexanol | 8000 | 0.0 | 0.0 | 0.0 | 0.0 | 0.0 | 0.0 |
| E-3-Hexen-1-ol | 400 | - | 0.0 | 0.0 | 0.0 | 0.0 | 0.0 |
| Z-3-Hexen-1-ol | 400 | 0.0 | 0.0 | 0.0 | 0.0 | 0.0 | 0.0 |

**Table S7:** Concentration of Acids, Alcohols, Aldehydes, C6 Compounds with highest OAV of wine samples fermented with *C. Boidini, C. Oleophila*and *C. Zemplinina* supplemented with DAP or YPH.

| COMPOUNDS | ODOR THRESHOLD (ppb) | Cb/DAP µg/L OAV** | Cb/YPH µg/L OAV** | Co/DAP µg/L OAV** | Co/YPH µg/L OAV** | Cz/DAP µg/L OAV** | Cz/YPH µg/L OAV** |
|---|---|---|---|---|---|---|---|
| ***Esters*** | | | | | | | |
| Diethyl butanedioate | 200000 | 0.0 | 0.0 | 0.0 | 0.0 | 0.0 | 0.0 |
| Ethyl 2-methylbutanoate | 18 | 1.0 | 1.2 | 0.1 | 0.1 | 0.2 | 0.2 |
| Ethyl 3-methylbutanoate | 3 | 6.3 | 7.6 | 0.7 | 0.9 | 0.7 | 0.8 |
| Ethyl 3-methylpentanoate | 0.5 | - | 3.8 | 1.6 | 3.2 | 1.4 | 2.6 |
| Ethyl Acetate | 12300 | 0.4 | 0.4 | 0.1 | 0.1 | 0.1 | 0.1 |
| Ethyl butanoate | 20 | 7.2 | 8.0 | 2.0 | 2.1 | 1.9 | 1.9 |
| Ethyl heptanoate | 220 | 0.0 | 0.0 | 0.0 | 0.0 | 0.0 | 0.0 |
| Ethyl hexanoate | 62 | 13.1 | 14.8 | 4.4 | 8.2 | 4.8 | 7.4 |
| Ethyl lactate | 154000 | 0.0 | 0.0 | 0.0 | 0.0 | 0.0 | 0.0 |
| Ethyl octanoate | 580 | 1.1 | 1.2 | 0.5 | 1.2 | 0.7 | 1.1 |
| Ethyl valerate | 94 | 0.0 | 0.0 | 0.0 | 0.0 | 0.0 | 0.0 |
| Hexyl ethanoate | 1500 | 0.0 | 0.0 | 0.0 | 0.0 | 0.0 | 0.0 |
| Isoamyl acetate | 30 | 21.9 | 22.8 | 9.7 | 10.0 | 8.3 | 12.3 |
| Isoamyl hexanoate | 30 | - | 0.1 | 0.1 | 0.1 | 0.1 | 0.1 |
| Isobutyl acetate | 1600 | 0.0 | 0.0 | 0.0 | 0.0 | 0.0 | 0.0 |
| Phenethyl acetate | 250 | 0.0 | 0.0 | 0.0 | 0.0 | 0.0 | 0.0 |
| ***Lactones*** | | | | | | | |
| Butyryl lactone | 35000 | 0.0 | 0.0 | 0.0 | 0.0 | 0.0 | 0.0 |

**Table S8:** Concentration of Esters, Lactones with highest OAV of wine samples fermented with *C. Boidini, C. Oleophila*and *C. Zemplinina* supplemented with DAP or YPH.
